# Long-term Follow-up Study for Fractured and Non-Fractured Hand Enchondromas Treated by Sole Curettage

**DOI:** 10.1055/a-2466-4905

**Published:** 2025-05-15

**Authors:** Cosima Prahm, Laura Kefalianakis, Johannes Heinzel, Jonas Kolbenschlag, Adrien Daigeler, Henrik Lauer

**Affiliations:** 1 Department of Hand, Plastic, Reconstructive and Burn Surgery, BG Klinik Tuebingen, University of Tuebingen, Germany

**Keywords:** enchondromas, hand tumors, radiological monitoring, bone augmentation, sole curettage

## Abstract

**Background** Enchondromas are the most common primary tumors in the small tubular bones of the hand, and fractures are often the result of thinned cortical bone. The main question was whether fractured enchondromas influence long-term clinical and radiological outcomes.

**Methods** Between 2000 and 2019, 57 patients with previously treated fractured (group I) and non-fractured (group II) hand enchondromas (34 female, 23 male; mean age 39.4 ± 13.7 years) were evaluated for clinical and radiological treatment outcomes. Short Form-36 Health Survey (SF-36) and Disabilities of the Arm Shoulder and Hand (DASH) questionnaires as well as patient-reported experience measures were used to assess subjective health outcomes. Subsequently, 43 patients underwent clinical and radiological follow-ups. Comparative evaluation of objective treatment outcomes in both groups was conducted in terms of hand functionality, perioperative complications, recurrence rates, and osteogenesis.

**Results** Almost half of the patients suffered enchondromas with fractures (49.1%, *n* = 28). Two patients received additional k-wire stabilization due to intraoperative instability. Defect resolution could be reached in 97.7% ( *n* = 42) of all cases. No recurrence of enchondroma was observed. Groups were equal regarding radiological and clinical outcomes. The patient-reported experiences were predominantly positive (86%), and both cohorts had good to very good results with a DASH mean score of 4 (± 6.3). The SF-36 demonstrated a return to normal quality of life in both groups. The mean follow-up time was 7.78 years (± 4.8).

**Conclusion** Sole curettage of enchondromas yields effective outcomes with good to excellent results regardless of the presence of a fracture. Long-term radiological follow-up is not required until symptomatic recurrence.

## Introduction

Enchondromas are the most common primary tumors in the small tubular bones of the hands. 1 They consist of hyaline cartilage and demonstrate slow-growing characteristics. 2 Even though enchondromas are benign tumors, they can compromise the structural integrity of bone tissue resulting in pathological fractures and further significant damage to the joints, ligaments, tendons, and neurovascular structures of the hand. 3

Treatment options for asymptomatic and symptomatic enchondromas without accompanying fractures are manifold. A large part of cases of hand enchondroma are discovered incidentally and are asymptomatic. 4 Regular radiological monitoring and conservative management are recommended for small localized asymptomatic lesions. 4 5

Surgical treatment involves tumor curettage with and without bone grafting. 4 6 Various bone graft materials have been described to fill enchondroma-evacuated cavities. 4 7 8 9 However, the use of autologous bone material may be associated with complications at the donor site, and from a financial perspective, the utilization of other bone graft materials lacks appeal. 4 7 Thereby, adjuvant treatments like high-speed burring or alcohol instillation are not recommended for enchondroma therapy, because similar outcomes are reached without additional actions. 4 10 11

Different approaches are discussed for pathological fractures. Depending on the presence of a displaced fracture, a one-stage treatment including curettage and placement of bone graft or bone block in addition to k-wire osteosynthesis or miniplates could be done. 12 Alternative option is to allow the fracture to heal first before curettage is performed. 4 Currently, there are no clear data available regarding the extent to which the presence of a fracture impacts the long-term healing process.

Recurrence rates of enchondromas after complete curettage is reported to be between 6 and 14%. 13 14 Malignant transformation seems to be rare, however, cases are described and especially the entity of chondrosarcoma appears difficult to differentiate. 1 9 15 16

In this study, we analyzed clinical and radiological outcomes in patients who received sole curettage for hand enchondroma. We explored the impact of pathological fractures on these outcomes and the necessity for specialized treatment protocols. The study compared enchondromas with and without fractures to evaluate treatment efficacy, posttreatment effects, and the relationship between tumor size and fracture risk. Additionally, we looked at the incidence of recurrence to see if curettage alone was sufficient to treat hand enchondroma. Our results were compared with historical data on the use of bone grafting or other adjuvant therapies.

## Methods

The study contains a retrospective data component and a prospective follow-up component. The inclusion criteria for the study applied to patients with hand enchondroma treated by sole curettage as identified in our database who were willing to answer questionnaires or undergo additional clinical and radiological follow-up examinations. The presence of a pathological fracture resulting from an enchondroma was not an exclusion criterion, as long as the treatment for the enchondroma consisted solely of curettage. A complete follow-up must have been completed at least 6 months ago. Patients had to be of legal age (18 years old) at the time of radiological follow-up. Exclusion criteria were bone augmentation or other additional treatments after curettage, and contraindications to radiographic imaging.

### Surgical Technique and Treatment Protocol

All surgical procedures were performed by specialists utilizing a dorsal approach. In instances of non-consolidated fractures, we confirmed fracture healing through x-rays and physical examination prior to the commencement of surgical intervention. If bony instability was observed postcurettage, the adjacent joints were immobilized for a period of 4 to 6 weeks. Following sole curettage, a postoperative regimen was implemented, ensuring that patients avoided weight-bearing activities for the first 2 weeks. Beyond this initial period, no specific movement restrictions were prescribed.

### Data Analysis

From 2000 to 2019, a retrospective study was conducted on all cases of hand enchondroma treated by sole curettage, confirmed through histological analysis. Patients were selected using diagnostic codes from our institutional database. Following selection, patients were contacted via telephone or mail to inquire about their willingness to participate in the study. Data recorded included gender distribution, age, duration of surgery, and length of follow-up. Information on follow-up treatments was documented based on available clinical records. Additionally, tumor size, potential recurrences, and any complications arising from the treatment were systematically recorded.

### Questionnaires and Clinical and Radiological Follow-up

Patients were asked to fill out the DASH questionnaire (Disabilities of the Arm Shoulder and Hand) and the SF-36 Health Survey (Short Form-36). 17 18 In addition, patient-related experience measures were collected by asking patients about their satisfaction with the treatment both during and after the procedure. Responses were classified into three categories: “very good,” “good/satisfied,” and “not satisfied.” During the follow-up study, the patients were called in to have their affected hands clinically and radiologically examined again. Radiographic examinations were performed by x-ray images in two perpendicular planes. The observer (H.L., J.K., A.D.) was blinded to the clinical outcome. The radiological image was inspected for postoperative osteogenesis and categorized according to the classification of Hasselgren et al. The classification involved four grades (grade I: excellent new bone formation, grade II: good new bone formation, grade III: scanty new bone formation, grade IV: no new bone formation). 19

As part of the physical examination, the sensitivity was assessed by static two-point discrimination. To objectively assess the operating result, an evaluation scheme quantifying movement restriction, sensory disturbances, pain, and cosmetic defects was applied, resulting in grade I (no limitations), grade II (mild limitations), and grade III (more severe limitations). 20

Clinical assessment and evaluation of radiological images were performed by senior physicians. The recurrence rate was recorded by the radiological follow-up examinations (X-rays) as well as patient history.

### Statistical Analysis

Patients presenting with pathological fractures due to hand enchondroma were categorized into Group I (fracture group), while those without fractures were classified into Group II (non-fracture group). These groups were analyzed to identify disparities in clinical and radiological outcomes. Statistical evaluations were conducted using Matlab R2022b (MathWorks Inc, MA). The normality of data was assessed with the Shapiro–Wilk test, followed by the application of either parametric or non-parametric tests based on the results. For normally distributed data, *t* -tests were employed, whereas the Mann–Whitney U test was utilized for data not following a normal distribution. A significance threshold was established at *p* < 0.05 for all statistical tests.

All patients who took part have been informed about the study in conformity with the Helsinki Declaration of 1975, as revised in 2008. Informed consent was obtained from all patients prior to inclusion in the study. The regional ethics committee approved this study under reference number 865/2019BO2.

## Results

During the study period, 170 patients underwent surgery for enchondromas of the hand. A total of 57 patients volunteered to participate in the study and completed all questionnaires (“fracture group,” group I: *n* = 28; “non-fracture group,” group II: *n* = 29). Of those, 43 agreed to participate in clinical and radiological follow-up (group I: *n* = 18, group II: *n* = 25). On average, follow-up for the clinical and radiological follow-up examinations and answering the questionnaires occurred 7.78 years after surgery, ranging from 2.1 to 18.9 years (mean value group I: 8.67 years; group II: 6.9 years).

**Fig. 1:**
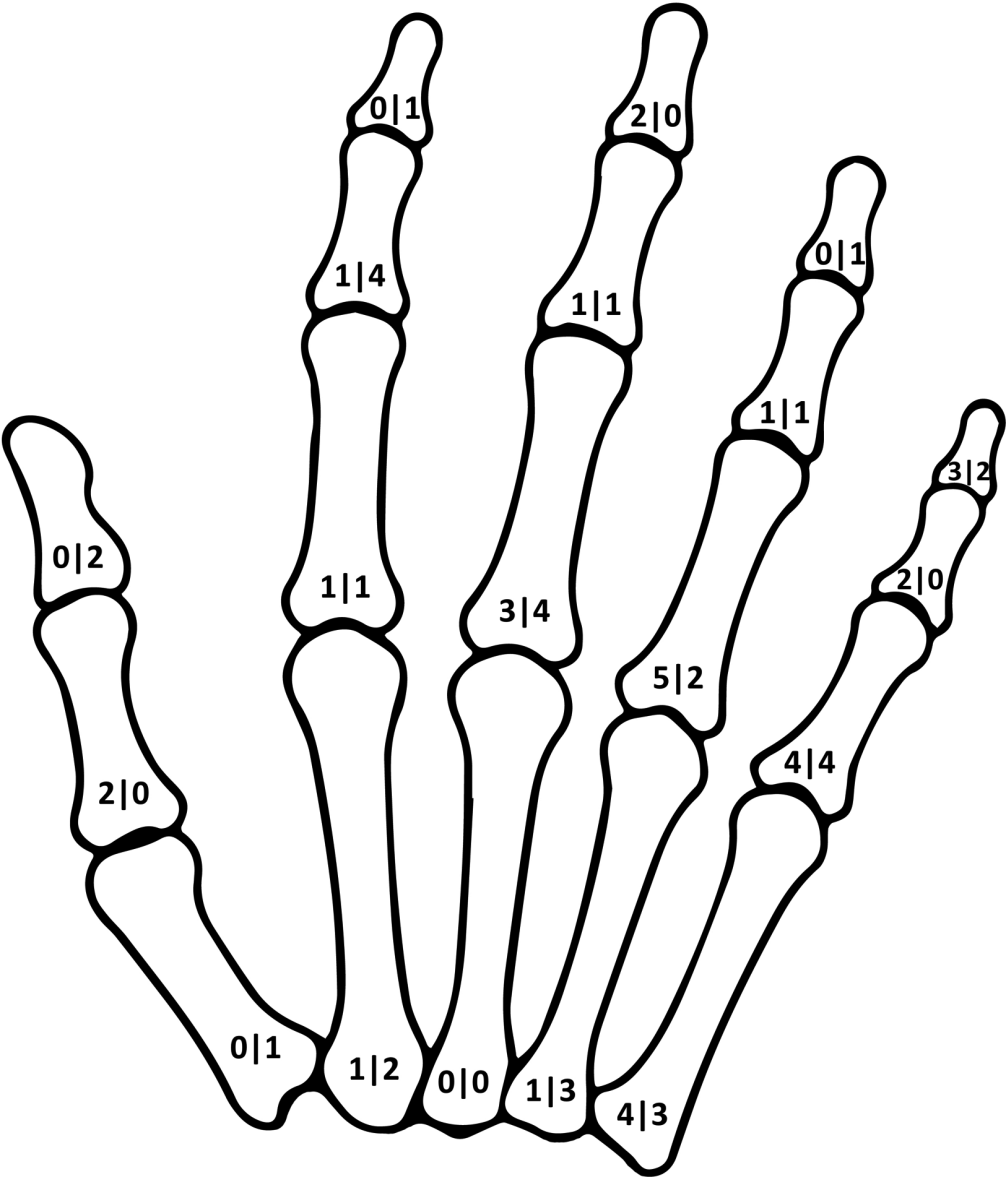
Schematic drawing showing the distribution of tumors on the respective phalanges and metacarpals (left side, number of enchondromas with fractures; right side, number of enchondromas without fractures).

In 54.4% ( *n* = 31) enchondromas affected the right hand. Two patients showed polyostotic, but unilateral tumors (in total, seven polyostotic tumors). None of these two patients could be classified as having Ollier disease or Maffucci syndrome. While the monostotic tumors mainly affected the fifth ray (35.1%, *n* = 20), the polyostotic tumors compromised predominantly the second ray (80%, Fig. 1 ). The proximal phalanx was mainly affected by 42.1% ( *n* = 24).

Three patients had been treated for previous enchondromas in other localizations at earlier times. In 25 out of 28 cases, fractured enchondromas were caused by minor trauma (89.3%). The most common symptoms of non-fractured enchondromas ( *n* = 29) were swelling or deformity in the affected area ( *n* = 20/29, 74.1%) followed by pain ( *n* = 7/20, 35%). Two patients were treated with curettage and an additional k-wire osteosynthesis because of intraoperative instability. No consolidated pathological fracture was noted at the time of the initial medical presentation. One patient developed a phlegmon of the dorsal hand after extirpation and required an additional intervention.

Tumor size in ulno–radial and proximal–distal extent did not differ in the groups of non-fractured enchondromas and fractured enchondromas ( *p* = 0.86). Table 1 shows a detailed list of the distribution and characteristics such as gender, age, operating time, and tumor size. A Mann–Whitney U test was used to compare the mean ages between the two groups, the *p* -value =0.2 indicates no significant difference in age between the groups. A chi-square test was used to compare the gender distribution between the two groups with *p* = 0.91, indicating no significant difference in gender distribution between the groups. These results confirm that the groups are comparable with respect to age and gender, validating their equivalence.

**Table 1:** Patient demographics and evaluation of patients with sole curettage

Participants	Enchondromas with pathological fractures	Enchondromas without pathological fractures	Total
Number of patients, *n*	28	29	57
Male/female	12 (42.9%)/16 (57.1%)	11 (40%)/18 (60%)	23 (40.4%)/34 (59.6%)
Mean age (SD), (years)	37.8 (± 12.6)	40.9 (± 15.6)	39.4 (± 13.7)
Follow-up (range), (years)	8.7 (0.7–18)	6.9 (0.3–19)	7.8 (0.3–19)
Operating time (range), (minutes)	30.6 (15–76)	31.7 (10–72)	31.2 (10–187)
Number of outpatients, *n*	23	26	49
Number of inpatients, *n*	5	3	8
Hospital stay (range), (days)	3.8 (2–7)	5 (3–8)	4.3 (2–8)
Suspected recurrence, *n*	0	0	0
Proximal–distal tumor size (SD), (mm) Radial–ulnar tumor size (SD), (mm)	15 (± 7.7) 9.5 (± 2.8)	15.2 (± 5.7) 10.7 (± 3.2)	15.1 (± 7) 10.2 (± 2.9)

Abbreviation: SD, standard deviation.

### DASH and SF-36 Health Questionnaire

**Fig. 2:**
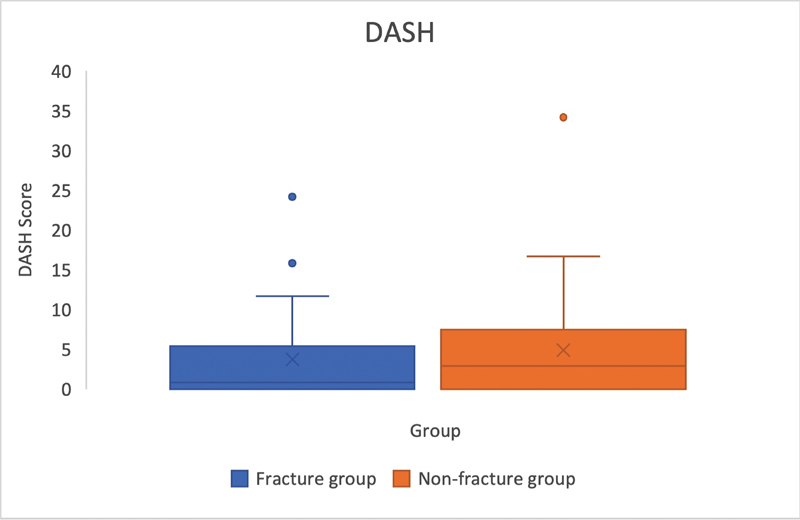
The DASH score for assessing upper extremity functionality including outliers and median, displayed for both groups (fracture group: 3 [±5.6]; non-fracture group: 5 [±7.3]). DASH, Disabilities of the Arm Shoulder and Hand.

**Fig. 3:**
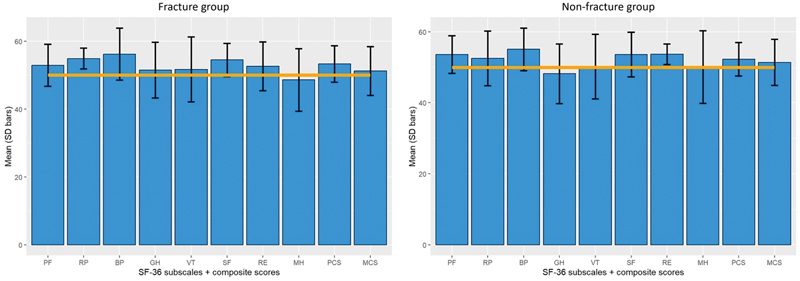
Normalized SF-36 subscale scores of enchondroma patients that presented with a fracture (left) or non-fracture (right) compared to the scores of the general healthy population (orange line, PF, physical functioning; RP, role physical; BP, bodily pain; GH, General Health; VT, vitality, SF, Social Functioning; RE, role-emotional; MH, mental health; PCS, Physical Composite Score; MCS, Mental Composite Score). SD, standard deviation; SF-36, Short Form-36 Health Survey.

All 57 patients had completed the DASH and SF-36 health questionnaires ( Figs. 2 and 3 ). The results of the DASH questionnaire showed a low impairment score for both groups. There were no significant differences between the groups ( *p* = 0.58). The SF-36 health questionnaire also showed no significant difference between the fracture and non-fracture groups regarding their physical ( *p* = 0.47) and mental composite scores ( *p* = 0.89). Moreover, both groups showed no significant differences compared to the healthy population and have returned to normal quality of life (fracture group: *p* = 0.77; non-fracture group: *p* = 0.69).

### Subjective Perception during Operative Treatment and Postoperative Course

According to the patient-related experience measures, 49 patients reported being very satisfied with the treatment, and outcomes were reported to be very good at 86% (49/57). Seven participants were moderately satisfied (8.1%).

### Patients for Clinical and Radiological Examinations

**Fig. 4:**
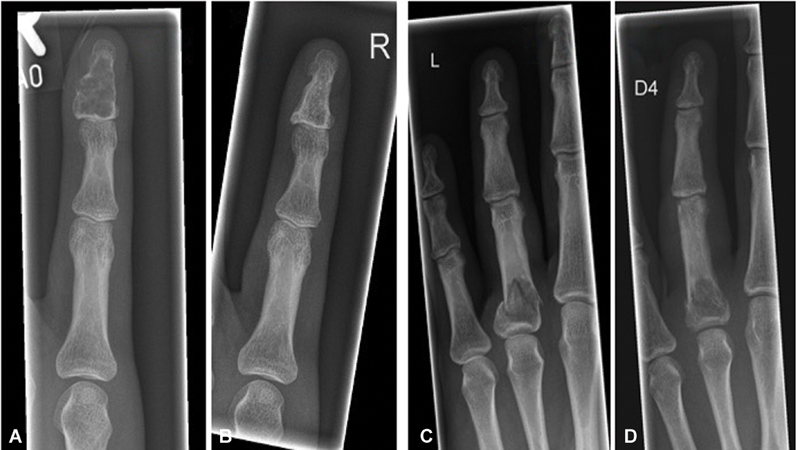
( **a** , **b** ) show the radiographs of a patient before and 6 years after curettage due to a non-fractured enchondroma at the distal phalanx of the little finger. ( **c** ) shows the X-ray image of another patient with a pathological fracture due to an enchondroma on the proximal phalanx of the ring finger. In this case, fracture healing was initially awaited. The enchondroma can be clearly demarcated after the fracture has healed ( **d** ).

Forty-three patients agreed in clinical and radiological follow-up examinations (75.4%; *n* = 43/57). Of these, 18 participants belonged to the fractured group (enchondromas with pathological fractures, 41.9%, female *n* = 10/male *n* = 8) and 25 participants to the non-fractured group (enchondromas without pathological fractures, 58.1%, female *n* = 15/male *n* = 10, Fig. 4 ). In total, 74.4% ( *n* = 32/43) of the patients had mild or no limitations in the criteria “Pain,” “Movement restrictions,” “Sensory disturbances,” and “Cosmetic defects” during the clinical examination. Twelve patients had more severe limitations (27.9%, Table 2 ). Of these 12 patients, 10 reported a difference in sensitivity compared to non-treated areas. Static two-point discrimination was in a normal range of 4.59 mm on average (± 2.1 mm). Complete loss of sensation or neuroma-associated symptoms did not occur. Three patients showed limitations of flexion in the adjacent finger joints (7%, *n* = 3/43). Four patients had measurable minor axis deviation to the opposite side (9.3%, 4/43). No significant difference could be found between both groups ( *p* = 0.39).

**Table 2:** Clinical assessment by evaluating the criteria “pain,” “movement restrictions,” “sensory disturbances,” and “cosmetic defects”

		Enchondromas with pathological fractures, *n*	Enchondromas without pathological fractures, *n*
Grade I	No limitations	5 (27.8%)	6 (24%)
Grade II	Mild limitations	9 (50%)	11 (44%)
Grade III	More severe limitations	4 (22.2%)	8 (32%)

### Radiological Results

Results of new bone formation are shown in Table 3 . Both groups displayed excellent and good new bone formation. The groups did not show any significant differences in outcomes ( *p* = 0.92). Bony healing was confirmed in all cases, and neither recurrences nor malignant transformations were identified during the radiological reevaluations.

**Table 3:** Evaluation of radiographic imaging

		Enchondromas with pathological fractures, *n*	Enchondromas without pathological fractures, *n*
Grade I	Excellent new bone formation in the cavity and normal cortical thickness	10 (55.6%)	14 (56%)
Grade II	Good new bone formation in the cavity and/or cysts <3 mm present	6 (33.3%)	7 (28%)
Grade III	Scanty new bone formation in the cavity and/or cysts >3 mm present	2 (11.1%)	4 (16%)
Grade IV	No new bone formation in the cavity	0	0

## Discussion

This study offers insights into the long-term outcomes of treating enchondromas with sole curettage by collecting data through patient questionnaires and conducting clinical and radiological follow-ups. The generally favorable results suggest that it is worth considering additional interventions, such as bone augmentation, for further discussion.

The present study is the largest report to date of long-term radiological and clinical follow-up of patients treated for hand enchondromas by curettage alone. Radiographic follow-up was performed for over 7 years and no evidence of suspected recurrence was observed. Although this study did not include interindividual comparison in our study, its findings are consistent with other studies that suggest that curettage alone does not lead to worse long-term outcomes. 6 10 21

The technique of sole curettage for hand chondromas has been well-documented. 19 22 However, additional bone augmentation is often deemed appropriate, particularly for large or expanding tumors. 9 In a study focusing on the metacarpus, Pianta et al illustrated that calcium phosphate bone cement enhanced strength compared to curettage alone in a cadaveric model of hand enchondroma. Nonetheless, they noted that in clinical practice, this added strength might not offer significant benefits. 23 Although artificial bone substitutes, including bioactive and osteoconductive materials in various forms, provide advantages such as reduced donor site morbidity and shorter operating times, their treatment cost is considerably higher compared to alternative options. 8 9 24 Autologous bone grafting is often considered the optimal choice due to factors such as avoidance of immune rejection, cost-effectiveness, and ready availability through access to the dorsal radius or pelvic region. However, there are two major concerns regarding bone augmentation. Firstly, the complication rate associated with the donor site in autologous bone transfer can be significant. For example, caution is advised when using iliac crest autografts for augmentation, as they may result in pain and infection at the donor site. 4 The main complications of distal radius bone grafting are pain, tenosynovitis, infection, fracture, and nerve injury. 25 Secondly, recurrence risk in solitary enchondromas does not appear to be positively affected by augmentation, 21 22 26 as recurrence is most likely caused by incomplete curettage. Due to the slow growth rates of these tumors, it can be challenging to detect suspected recurrences, and additive bone grafting does not improve outcomes after surgical therapy for solitary enchondroma in the hand. 6 22 26

In our opinion, autologous bone grafting continues to play a significant role in the treatment of enchondromas. During the study period at our center, 6.5% of all patients with enchondromas (11 out of 170) presented with a massive cortical defect and intraoperative instability. Due to the severity of these defects, these patients were treated with autologous bone grafts harvested from the iliac crest. Of these patients, eight underwent additional osteosynthesis or arthrodesis procedures. Six patients reported experiencing pain at the donor site for several weeks; however, this discomfort resolved by the seventh postoperative week at the latest. All patients demonstrated successful consolidation and expressed general satisfaction with their outcomes. It is important to note that long-term results remain undetermined due to these patients not being part of the inclusion criteria.

Consistent with findings from other studies, our study population predominantly exhibited diagnosis establishment in the fourth decade of life, with no gender specificity. 27 Enchondromas were most localized at the proximal and ulnar parts of the phalanx, aligning with existing literature. 27 Within our study, two patients presented with polyostotic tumors. In both cases, the tumors affected only one hand unilaterally, with no other tumors detected, suggesting a possible diagnosis of Ollier disease, also known as multiple cartilaginous enchondromatosis. While malignant transformation of one or more enchondromas towards secondary central chondrosarcoma is possible, such transformations typically affect enchondromas of long tubular and flat bones, with the risk of malignant transformation in hand enchondromas being low. 28 Notably, these two patients exhibited no changes in tumor deformity and experienced no recurrence following sole curettage.

Most enchondromas were diagnosed because of symptoms and complaints such as swelling, impaired hand function, and/or pain in the affected area. In our patient cohort, 93% of those with non-fractured tumors presented with these symptoms. Pathological fractures are commonly feared during the progression of the disease and occur in up to two-thirds of patients. 29 Because of the bone's weakened state, even minor trauma can result in fracture. In our patient cohort, approximately half experienced fractures, with the majority of cases attributed to minor traumas (89.3%). These observations align with findings reported in the literature. 14

Riester et al tried to identify objective and reproducible criteria that predict a patient's risk of developing a pathological fracture due to an enchondroma. 3 They determined that younger age, smaller finger size, localization in the distal and proximal phalanx, and particularly the percentage of bone occupied by the tumor in the longitudinal dimension are correlated with a greater risk of developing fractures. 3 In our study, we did not observe a difference in tumor size between fractured and non-fractured enchondromas. While the concept of greater loss of bone integrity leading to increased fracture risk seems reasonable, cortical thinning appears to be more plausible for pathological fractures. 3 However, in our study, pathological fractures caused by enchondromas were permitted to heal before curettage was performed. Only two patients underwent treatment with k-wire osteosynthesis due to intraoperative instability resulting from curettage.

The overall outcome of surgical treatment for fractured enchondromas is expected to be largely favorable, with outcomes typically ranging from good to very good and rare complications, similar to those observed in non-fractured enchondromas. 12 In a systematic review by Tang et al of mainly level IV studies, complication rates ranged from 0 to 3.5%. 4 Regardless, significant malalignment should be corrected promptly. If the fracture necessitates an open approach, immediate treatment of the enchondroma should be considered. 30 Some authors prefer bone augmentation in fractured enchondromas, as they consider cortical thinning due to curettage a risk to subsequent bone healing. 24 However, there are currently no long-term results that have been radiologically verified. In our study, patients were followed up for 7 years, and the use of curettage alone did not result in worse outcomes in terms of bone healing and clinical outcomes for fractured enchondromas.

Within the scope of this study, patients reported high satisfaction following surgical treatment, which corresponded with their perception of upper extremity health status and function. Both groups exhibited a median DASH questionnaire score below 6, indicating high self-reported functionality in hand and finger movements ( Table 1 and Fig. 2 ). This suggests that patients were capable of performing most daily activities without notable difficulty or impairment. These results align with those of the SF-36 questionnaire, which revealed no significant differences from a healthy norm population.

A conservative approach as an alternative treatment demonstrated a significantly better functional outcome in comparison to operative procedures, as shown by a larger study investigating the outcome of enchondromas and atypical cartilaginous tumors of the long bones. 31 The generalizability of these findings to enchondromas of the hand is uncertain and may be subject to questioning. It is undeniable that the indication for operative treatment of enchondromas is given if specific symptoms are present. 20 In case of a non-symptomatic enchondroma and small extension as well as lacking signs of cortical damage clinical follow-up examinations are justifiable.

This study has some limitations. Firstly, the patient population did not receive alternative treatment options such as bone augmentation, which limited these comparisons to existing literature. The use of bone augmentation is of relevance, especially in cases of defects with extensive cortical destruction. However, despite this limitation, the study revealed a low complication rate and generally favorable to very favorable outcomes, even in cases involving larger hand enchondromas with no observed recurrences. Secondly, the study design permitted only a single assessment of clinical and radiological outcomes years after surgery, and a quarter of the patients declined clinical and radiological follow-up examinations. Moreover, these assessments were not conducted at strictly defined intervals postoperation, except for a requirement of at least 6 months. Therefore, patient-perceived insight into the immediate postoperative course was limited and the comparison between the two groups must be viewed critically. Due to the extended follow-up period, there may be a bias in the patient's personal perception of their outcomes. However, it can be stated that patients with pathological fractures due to hand enchondromas do not have to expect a more complicated healing process.

### Conclusion

Sole curettage demonstrates a low rate of complications and is generally well-accepted as a treatment for hand enchondromas. Recurrence is rare, and patient satisfaction is high. Our study indicated that the presence of pathological fractures did not impact long-term outcomes. Curettage alone proves to be adequate as a therapy for enchondromas, even in cases of pathological fractures. However, it is advisable to allow fracture healing to occur first. In the case of extensive cortical destruction, cancellous bone surgery should nevertheless be discussed as an additional therapeutic procedure.
